# The Elimination of the Middle Classes

**Published:** 1913-12-27

**Authors:** 


### THE EDITOR'S LETTER-BOX.

### The Elimination of the Middle Classes.

*To the Editor of* The Hospital.

Sir,—A writer, who claims to be the mother of three children, of the middle class wrote recently to a newspaper to say that in her despair at the difficulty of making ends meet she is often inclined to throw herself and her children into the river. Assuredly at the present time things are hard upon people of moderate income. With incomes which do not exceed and sometimes do not reach that of a skilled artisan, they have to maintain a position above that of the working classes. The cost of living increases, but, at least in the middle class, incomes do not. And, at the same time, middle-class parents are reproached for not setting a good example to the community by having large families. There is no doubt that parents with a sense of parental responsibility are inclined to limit their families, either by postponing the age of marriage or by other means. They consider the cost of feeding and clothing children sufficiently, educating them suitably, and finally setting them out in the world, and so they hesitate. The people who do not consider do not hesitate, and thence come endless appeals for help. It is no wonder that the middle classes begin to think that they are exploited for the benefit of those both richer and with less self-control and forethought than themselves. Imperial taxation catches them in many ways, and the Poor Law and education rates tax them locally. To ask them to increase their expenditure on their families is to ask the impossible. One thing they can do, and one thing they will certainly be forced to do before long, is to take advantage of the opportunities which the State puts before them in the education of their children. In America, in Germany, even in Scotland, people of decent position send their children to the State-supported schools. They contribute to the maintenance of these schools, they do not grudge a share in their advantages to their fellow-citizens of whatever class, and why should they not benefit by the good buildings, good apparatus, and good teachers which their funds supply? With education provided for, many parents could face the prospect of a larger family than they will think of now. The education is there. Will they profit by it?—Yours faithfully, F. S.

[Few subjects excite more controversy to-day than the decline in the birth-rate. Our own views have been expressed recently. But here is a particular aspect on which our readers' experience would be invaluable.—Ed., The Hospital.]

